# Intracholecystic Papillary Neoplasm With Associated Invasive Carcinoma, Accompanied by Tumor Thrombi of Hepatoid Adenocarcinoma: A Case Report

**DOI:** 10.1155/crip/7736309

**Published:** 2025-10-03

**Authors:** Sho Yoshida, Yusuke Kouchi, Ryotaro Eto, Masayuki Ohtsuka, Takashi Kishimoto

**Affiliations:** ^1^Chiba University School of Medicine, Chiba, Japan; ^2^Department of Molecular Pathology, Chiba University Graduate School of Medicine, Chiba, Japan; ^3^Department of General Surgery, Chiba University Graduate School of Medicine, Chiba, Japan

**Keywords:** gallbladder, hepatoid adenocarcinoma, intracholecystic papillary neoplasm, SALL4, tumor thrombi

## Abstract

Hepatoid adenocarcinoma (HAC) is a rare extrahepatic adenocarcinoma characterized by hepatocellular differentiation. HAC arising in the gallbladder is rare. We report a case of intracholecystic papillary neoplasm (ICPN) with HAC, most of which were tumor thrombi. The patient was a 78-year-old Japanese woman diagnosed with a gallbladder mass, detected by ultrasonography. She underwent cholecystectomy with lymph node dissection. Histopathological examination revealed exophytic papillary growth consistent with ICPN with associated invasive carcinoma. Notably, many tumor nests displayed characteristics of HAC, including eosinophilic granular cytoplasm and solid trabecular patterns. Immunohistochemically, tumor cells were positive for AFP, Glypican 3, and Hep Par 1 but negative for SALL4. Our findings underscore the importance of recognizing HAC components in gallbladder lesions, particularly in the context of ICPN with associated invasive carcinoma, as they may significantly impact patient prognosis.

## 1. Introduction

Hepatoid adenocarcinoma (HAC) is an extrahepatic adenocarcinoma with hepatocellular differentiation, showing histological resemblance to hepatocellular carcinoma and producing plasma proteins such as AFP [1]. While the stomach is the most common site of occurrence, HAC can also develop in various organs, including the pancreas [2], colon [3], and lung [4]. HAC arising in the gallbladder is rare, and to our knowledge, only 23 cases of HAC in the gallbladder have been reported in the English literature [5–13].

Intracholecystic papillary neoplasm (ICPN) is a newly classified in the WHO classification fifth edition in 2019 [14], defined as a noninvasive epithelial neoplasm forming a grossly visible mass that protrudes into the lumen of the gallbladder. It is considered the counterpart of pancreatic intraductal papillary mucinous neoplasm and intraductal papillary neoplasm of the bile ducts. Invasive carcinoma is observed in approximately half of ICPN cases and is more common in the areas with a predominant biliary phenotype and/or with high-grade dysplasia [14].

We report a rare case of ICPN with associated invasive carcinoma, in which the predominant part of the HAC components was presented as tumor thrombi.

## 2. Case Presentation

A 78-year-old Japanese woman was referred to our hospital for further examination and treatment of a gallbladder mass detected with an abdominal ultrasonography by her local physician. Her medical history included hypertension, asthma, total shoulder arthroplasty, and a right femoral neck fracture. She had no significant family history, and she does not smoke or drink alcohol. No abnormalities were found at the physical examination. The levels of serum CA19-9 and CEA were 40.4 U/mL and 5.9 ng/mL, respectively. Serum AFP level was not tested. Liver function tests showed slight abnormalities (AST, 51 U/I; ALT, 47 U/I; and *γ*-GTP, 67 U/I). Hepatitis B virus surface antigen and hepatitis C virus antibody were negative. Abdominal dynamic computed tomography (CT) showed that a broad-based mass measuring 36 mm in major diameter occupied the fundus to body of the gallbladder. There was no finding that suggested metastasis of the lymph node or other organs including the liver. Whole-layer cholecystectomy with gallbladder bed resection and lymph node dissection was performed.

**Figure 1:**
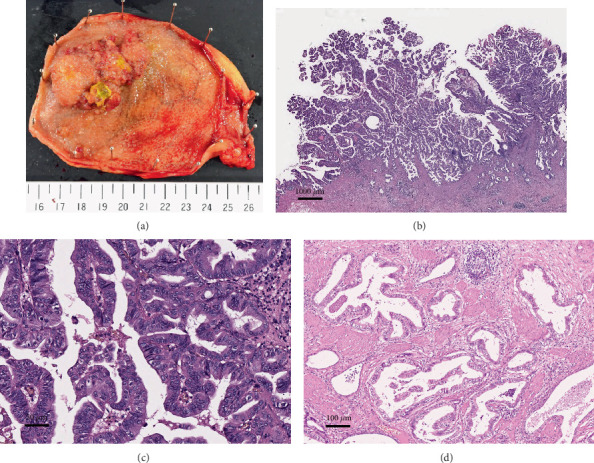
Macroscopic appearance of the cholecystectomy specimen. (a) A 55 × 35 mm papillary tumor protruding into the lumen, extending from the body to the fundus of the gallbladder. (b) The tumor predominantly exhibited a papillary growth pattern with a narrow stroma (4×). (c) The tumor cells were cuboidal with eosinophilic cytoplasm and enlarged nuclei, consistent with the biliary morphology with high-grade dysplasia (200×). (d) The associated well-differentiated adenocarcinoma had invaded to the subserosal layer (100×).

Macroscopically, a granular-surfaced, polypoid lesion measuring 55 × 35 mm was observed extending from the fundus to the body of the gallbladder (Figure 1a).

**Figure 2:**
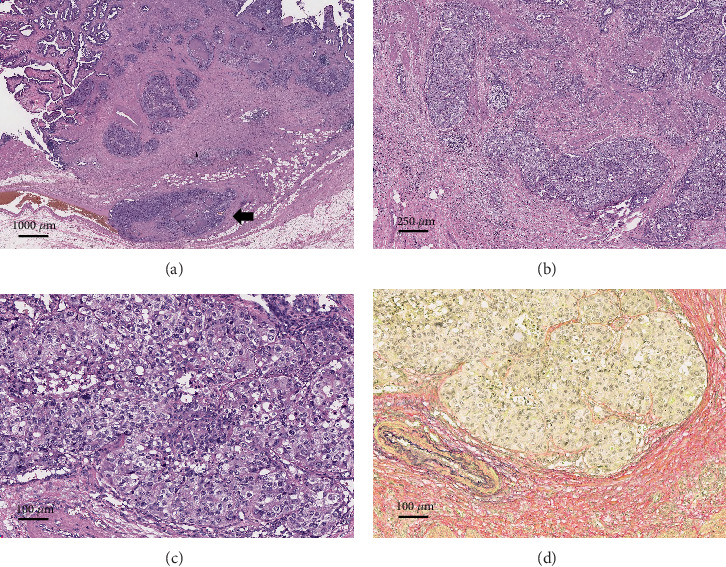
Components of hepatoid adenocarcinoma. Well-circumscribed nests were observed in the gallbladder wall. (a) Note the large tumor thrombus (arrow) (4×). (b) The solid nests with no distinct glandular structure (40×). (c) The tumor cells had eosinophilic finely granular or clear cytoplasm and enlarged nuclei, arranged in a trabecular pattern (100×). (d) Elastic fiber stain (EVG) revealed that the nests were intravascular tumor thrombi (100×).

**Figure 3:**
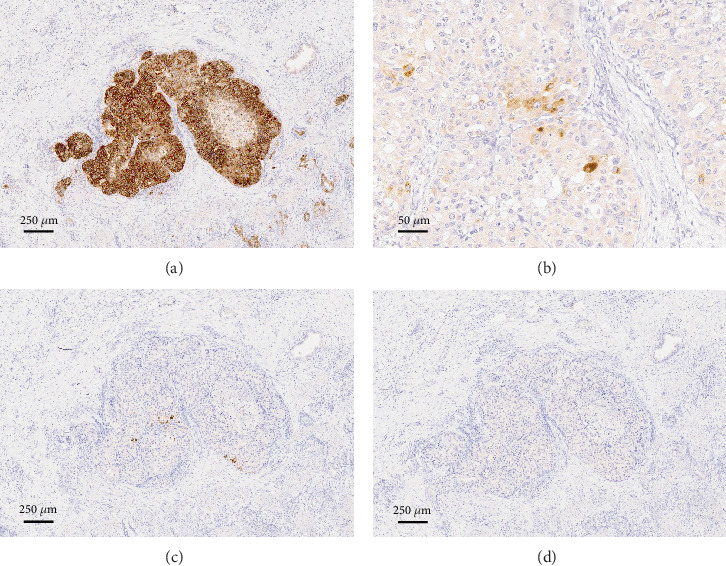
Immunohistochemistry of the intravascular tumor nests was positive for (a) Glypican 3 (40×), partially positive for (b) AFP (200×) and (c) Hep Par 1 (40×), but negative for (d) SALL4 (40×).

Microscopically, the tumor exhibited exophytic growth with a predominantly papillary configuration. The stroma was narrow and exhibited a fern-like structure in part (Figure 1). The tumor cells were cuboidal to columnar with moderate to severe nuclear atypia (Figure 1). These histological findings were consistent with ICPN (Figure 2). Associated invasive carcinoma of well-differentiated tubular adenocarcinoma infiltrated down to the subserosal layer (Figure 1). Several well-defined tumor nests were observed in the invasive area, characterized by tumor cells with eosinophilic, granular, and relatively abundant cytoplasm proliferating in a solid trabecular pattern without forming glandular structures. The features were similar to those of hepatocellular carcinoma (Figures 2, 2, and 2). Observation with EVG staining revealed that many of the nests were tumor thrombi (Figure 2). Immunohistochemically, these tumor cells were partially positive for AFP and Hep Par 1 and diffusely positive for Glypican 3 (GPC3), but SALL4 was negative (Figure 3). On the basis of histological and immunohistochemical findings, these nests were determined to be HAC.

The tumor cells of ICPN were partially positive for MUC1, MUC6, and CDX2 but negative for MUC2 and MUC5AC. The tumor cells of the hepatoid component showed diffuse and strong positivity for p53, suggesting a *TP53* gene mutation. SMAD4 expression was retained in all tumor cells.

Chemotherapy was not administered as it was not desired by the patient and her family. Five months after surgery, a CT scan revealed multiple liver metastases. She is still alive 16 months after the surgery.

## 3. Discussion

HAC was first reported by Ishikura et al. in 1985 as a special subtype of gastric adenocarcinoma [1]. HAC is rare in the gallbladder, with only 23 cases reported to date [5–13]. The mean age of patients is 67.1 years (ranging from 52 to 80), with a slight female predominance (F/M, 13/9). Most cases originate from the body to the fundus of the gallbladder (Table 1). We reported a case of ICPN with associated invasive carcinoma with HAC. Notably, many of the HAC were presented as intravascular tumor thrombi. While ICPN may be a relatively new classification [14], there have been no previous reports of HAC in ICPN with associated invasive carcinoma. However, the case of combined hepatocholangiocarcinoma arising in ICPN, reported by Dettoni et al. in 2014, may represent the first documented instance [15].

In addition to its histological morphology, HAC exhibits various hepatocytic features, which are thought to result from the hepatic transdifferentiation of tubular adenocarcinoma [16]. Although the molecular mechanism of HAC development has not been elucidated, in vitro studies have demonstrated that the introduction of hepatocyte-specific transcription factors can induce hepatic transdifferentiation in adenocarcinoma cells [17].

Clinically significant is the fact that HAC is a highly malignant subtype. It shows strong vascular invasiveness, leading to early hepatic metastasis and recurrence, with a very poor prognosis [18]. Additionally, it is resistant to chemotherapy, further contributing to its poor prognosis [19]. In our case, a significant portion of the HAC component was tumor emboli within the vessels. While ICPN with associated invasive carcinoma generally has a better prognosis compared to typical gallbladder cancer, the presence of even a small HAC component may negatively impact the prognosis.

The histological appearance of HAC is characterized by its similarity to hepatocellular carcinoma. Typically, cuboidal cells with abundant eosinophilic, finely granular cytoplasm proliferate in medium to thick trabecular structures. Areas of conventional tubular adenocarcinoma are often intermixed, with transitional features observed between the two. Clear cells with cytoplasmic glycogen accumulation may also be present. HAC is primarily diagnosed based on its distinctive histological features, but immunohistochemical staining can assist in diagnosis, with positivity for AFP, SALL4, GPC3, and Hep Par 1 [20]. In gallbladder HAC cases studied to date including the present case, AFP was positive in 10 out of 16 cases, GPC3 in 5 out of 7, and Hep Par 1 in 11 out of 12 cases. While SALL4 is typically positive in gastric HAC [20], all three gallbladder HAC cases examined, including ours, were negative for SALL4 [7] (Table 1). Although the number of cases analyzed is small, the relatively higher frequency of AFP-negative or SALL4-negative cases warrants attention.

## 4. Conclusion

We reported the first case of ICPN-associated invasive carcinoma with a HAC component. Notably, a significant portion of the HAC component was found as tumor thrombi. Although HAC was not the dominant component, it is considered an important finding that may impact the prognosis.

## Figures and Tables

**Table 1:** Clinicopathologic features of hepatoid adenocarcinoma of the gallbladder.

**Case**	**Age**	**Sex**	**Symptom**	**Location**	**Size (mm)**	**AFP (pg/mL)**	**Prognosis**	**Immunohistochemistry**				**Ref**
								**AFP**	**HepPar1**	**GPC 3**	**SALL4**	
1	52	F	N/A	N/A	40 × 30	17,363	12 months DOD	N/A	N/A	N/A	N/A	[5]
2	66	F	N/A	N/A	70 × 30 × 20	31,467	N/A	+	N/A	N/A	N/A	[6]
3	77	M	General fatigue, weight loss	F	45 × 40 × 37	3.5	15 months, NED	−	N/A	N/A	N/A	[8]
4	72	M	Abdominal pain	B	60 × 30 × 30	N/A	5 months DOD	−	+	N/A	N/A	[8]
5	74	F	N/A	B	65 × 40 × 35	N/A	N/A	+	+	N/A	N/A	[8]
6	74	F	N/A	B	50 × 45 × 18	N/A	N/A	−	+	N/A	N/A	[8]
7	55	F	Abdominal pain	F	70	511	N/A	+	N/A	N/A	N/A	[8]
8	76	F	Abdominal pain, fatigue	N	25 × 15 × 5	N/A	8 months, NED	+	+	N/A	N/A	[8]
9	71	F	Abdominal pain	N/A	40 × 30	N/A	1 month, DOO^a^	+	N/A	N/A	N/A	[8]
10	59	F	Abdominal pain, fatigue	B	110 × 50	N/A	3 months, NED	+	+	N/A	N/A	[8]
11	60	F	Fever, fatigue	B	60 × 50 × 30	62	20 months	+	N/A	N/A	N/A	[8]
12	61	M	N/A	B	60 × 30 × 30	173.55	5 months, DOD	+	−	N/A	N/A	[8]
14	43	M	Abdominal pain	F to N	120 × 50 × 20	23,500	1 month, alive	+	+	N/A	N/A	[8]
15	80	M	Abdominal pain	B	46 × 56 × 71	2.46	19 months, NED	−	+	+	N/A	[8]
16	64	M	N/A	N/A	N/A	N/A	3 months	−	N/A	+	−	[7]
17	75	M	N/A	N/A	N/A	N/A	37 months, alive	−	N/A	−	−	[7]
18	80	F	Back pain, weight loss	F and B	50 × 40	N/A	3 months, NED	N/A	+	−	N/A	[8]
19	61	M	N/A	F	32 × 23	Normal	5 months, alive	N/A	N/A	N/A	N/A	[8]
20	67	F	Abdominal pain	N/A	N/A	Elevated	N/A	N/A	N/A	N/A	N/A	[9]
21	69	F	Abdominal distention, constipation	N	38 × 35 × 10	Normal	5 months, NED	N/A	+	+	N/A	[10]
22	82	F	N/A	B	25 × 23	N/A	9 months, AWD	N/A	+	N/A	N/A	[11]
23	60	M	N/A	N/A	N/A	42	N/A	N/A	N/A	+	N/A	[12]
Our case	78	F	None	F to B	35 × 55	N/A	16 months, AWD	+	+	+	−	

Abbreviations: AWD, alive with disease; B, body; DOD, dead of disease; F, fundus; GPC3, Glypican 3; N, neck; N/A, data not available; NED, no evidence of disease; Ref, reference number.

^a^Died of renal failure.

## Data Availability

The data that support the findings of this study are available from the corresponding author upon reasonable request.
